# Bioinformatic analysis of the fold type I PLP‐dependent enzymes reveals determinants of reaction specificity in l‐threonine aldolase from *Aeromonas jandaei*

**DOI:** 10.1002/2211-5463.12441

**Published:** 2018-05-21

**Authors:** Kateryna Fesko, Dmitry Suplatov, Vytas Švedas

**Affiliations:** ^1^ Institute of Organic Chemistry Graz University of Technology Austria; ^2^ Belozersky Institute of Physicochemical Biology Lomonosov Moscow State University Russia

**Keywords:** bioinformatics, enzyme catalysis, protein engineering, pyridoxal‐phosphate, threonine aldolase

## Abstract

Understanding the role of specific amino acid residues in the molecular mechanism of a protein's function is one of the most challenging problems in modern biology. A systematic bioinformatic analysis of protein families and superfamilies can help in the study of structure–function relationships and in the design of improved variants of enzymes/proteins, but represents a methodological challenge. The pyridoxal‐5′‐phosphate (PLP)‐dependent enzymes are catalytically diverse and include the aspartate aminotransferase superfamily which implements a common structural framework known as type fold I. In this work, the recently developed bioinformatic online methods Mustguseal and Zebra were used to collect and study a large representative set of the aspartate aminotransferase superfamily with high structural, but low sequence similarity to l‐threonine aldolase from *Aeromonas jandaei* (LTAaj), in order to identify conserved positions that provide general properties in the superfamily, and to reveal family‐specific positions (FSPs) responsible for functional diversity. The roles of the identified residues in the catalytic mechanism and reaction specificity of LTAaj were then studied by experimental site‐directed mutagenesis and molecular modelling. It was shown that FSPs determine reaction specificity by coordinating the PLP cofactor in the enzyme's active centre, thus influencing its activation and the tautomeric equilibrium of the intermediates, which can be used as hotspots to modulate the protein's functional properties. Mutagenesis at the selected FSPs in LTAaj led to a reduction in a native catalytic activity and increased the rate of promiscuous reactions. The results provide insight into the structural basis of catalytic promiscuity of the PLP‐dependent enzymes and demonstrate the potential of bioinformatic analysis in studying structure–function relationship in protein superfamilies.

**Abbreviations**
FSP: family‐specific positions
LTA: l‐threonine aldolase
PLP: pyridoxal‐5′‐phosphate

Pyridoxal‐5′‐phosphate (PLP), the active form of vitamin B6, is a versatile cofactor used by more than 160 different enzymatic reactions classified by the Enzyme Commission. PLP‐dependent enzymes are mainly involved in the amino acid metabolism in all living organisms and catalyse a number of diverse chemical reactions, such as decarboxylation, transamination, racemization, β‐elimination, carbon‐carbon bond cleavage and formation 1, 2. Due to their catalytic versatility, the PLP‐enzymes have been exploited as biocatalysts for the production of natural and non‐natural amino acids and amino acid derivatives 3. The aspartate aminotransferase superfamily is the largest group of PLP‐dependent enzymes which implement versatile catalytic activities (aldolase, transaminase, decarboxylase, etc.) within a common structural framework commonly being referred to as the Fold type I 4. l‐threonine aldolases (LTAs) are members of the aminotransferase superfamily and catalyze the stereoselective reversible retro‐aldol cleavage of l‐threonine and a range of l‐β‐hydroxy‐α‐amino acids 5, 6. LTAs attract increasing attention as biocatalysts for the asymmetric carbon‐carbon bond formation to synthesize β‐hydroxy‐α‐amino acids from achiral aldehydes and glycine (Fig. 1) 7. Enzymes of this family are highly selective at the α‐carbon of l‐threonine and are classified into l‐*allo*‐ and l‐*low‐specificity*‐TAs based on their selectivity at the β‐carbon. The structural organization, catalytic mechanisms, synthetic and biochemical properties of microbial LTAs have been recently reviewed 8, 9.

**Figure 1:**
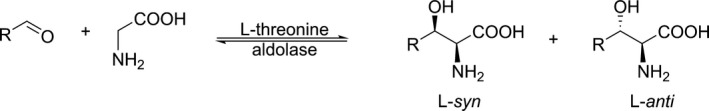
Aldol synthesis of l‐β‐hydroxy‐α‐amino acids catalysed by LTA.

The catalytic diversity of the PLP‐dependent enzymes within a common structural fold is investigated by various research groups in an attempt to improve our understanding of the structure‐function relationship in these proteins and to design improved enzymes for biotechnological application. The catalytic mechanism of aspartate aminotransferase superfamily enzymes is depicted in Fig. 2. According to the Dunathan's theory, the stereoelectronic effect is the major determinant of the reaction type in a PLP‐dependent enzyme, i.e. the bond at C_α_ of the external aldimine to be cleaved should be perpendicular to the pyridine ring of the cofactor, and thus the active site should be evolved to bind the substrate in a correct initial orientation (Fig. 2, external aldimine) 10, 11. The majority of conversions catalysed by aspartate aminotransferase superfamily enzymes begin with the abstraction of a proton from the C_α_ atom of the substrate (e.g. in cystathionine β‐lyase, transaminase, kynureninase, racemase). In a contrast, the retro‐aldol reaction catalysed by LTA is initiated by the C_α_–C_β_ bond cleavage following the deprotonation of a hydroxyl group at C_β_ of the l‐β‐hydroxy‐α‐amino acid substrate. The initiation step is followed by the formation of a carbanionic intermediate, which is stabilized by the delocalization of the negative charge in the PLP ring (Fig. 2, quinonoid). Final protonation of the quinonoid intermediate can occur either at C_α_ of aldimine or at C_4_′ of the PLP leading to different products. Thus, it was suggested that the particular catalytic pathway – i.e., the reaction specificity – within the superfamily is defined by a specific set of amino acids at the key positions in the enzyme's active site, which ensure, at first, that a particular bond at C_α_ of the external aldimine is correctly oriented for cleavage, and then define the protonation site to release the product. The amino acid residues which play a key role in determining the reaction specificity in the aspartate aminotransferase superfamily were not studied systematically so far. The molecular mechanisms that control the catalytic preference were investigated in individual PLP‐dependent enzymes 1, 11, and in the class III transaminases 12, however, LTAs have not been previously subjected for the analysis.

**Figure 2:**
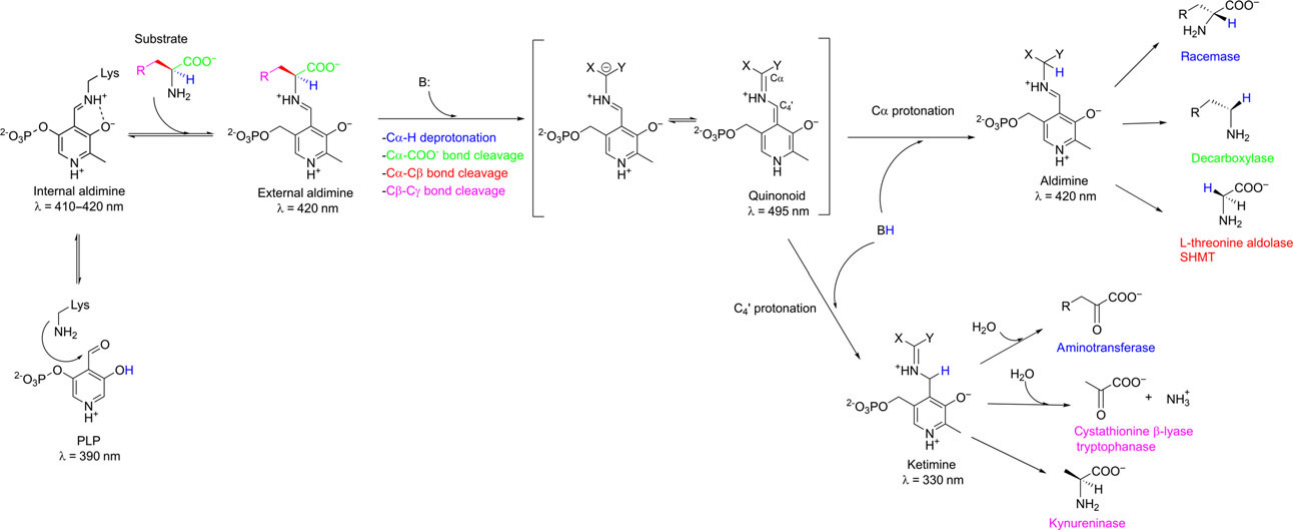
Mechanism of catalysis by the enzymes from the aspartate aminotransferase superfamily.

Recent years have seen a general trend away from the inefficient stochastic approaches (e.g., directed evolution) towards more rational and focused strategies to study the structure‐function relationship in proteins and design novel enzymes 13, 14, 15. In this context, the methods of bioinformatics attract increasing attention as they provide an opportunity to systematically study enzymes by comparative analysis of the growing amount of sequence and structural data. Multiple alignment of homologous proteins presents an opportunity to study functionally important residues by understanding their sequence variability patterns within a superfamily 12, 13, 16, 17, 18, 19. However, construction and further bioinformatic and statistical analysis of large superfamily alignments that can include thousands of proteins require specialized software and skills. The user‐friendly 3DM tool to study the structure‐function relationship in large alignments of homologous proteins was developed, but the publicly available content is limited, and the access is generally provided on a commercial basis 12, 20. Thus, integration of bioinformatic analysis with experimental techniques is slower than expected, and stochastic approaches remain the most widely used methods in protein design and biotechnology as of today 21. Two bioinformatic methods – Mustguseal 22 and Zebra 23 – have been recently developed to study the structure‐function relationship in proteins and are freely available on‐line. The Mustguseal web‐server can automatically construct large structure‐guided sequence alignments of functionally diverse protein families/superfamilies basing on all available information about their structures and sequences in public databases. Integrated sister web‐server Zebra can be further used to analyse the obtained alignment. In this work, a structure‐guided sequence alignment of the Fold type I PLP‐dependent enzymes has been constructed by the Mustguseal method with a particular focus on LTAs to compare the evolutionarily distantly related enzymes implementing different reaction specificities within a common structural framework. The bioinformatic analysis by the Zebra method has been further used to identify conserved positions in protein structures which define the general properties in the aminotransferase superfamily, as well as family‐specific positions (FSP) to reveal the reaction specificity determinants. The implication of positions identified by the bioinformatic analysis to function and catalytic activity of LTA from *Aeromonas jandaei* (LTAaj) then has been studied by experimental site‐directed mutagenesis and molecular modelling. This study provided a comprehensive set of amino acid residues important for the retro‐aldol and aldol transformations and the reaction specificity in LTAaj. Enzyme variants with novel catalytic activity have been produced by introducing mutations at selected positions identified by the bioinformatic analysis.

## Results and Discussion

### Bioinformatic analysis of the aminotransferase superfamily

**Figure 3:**
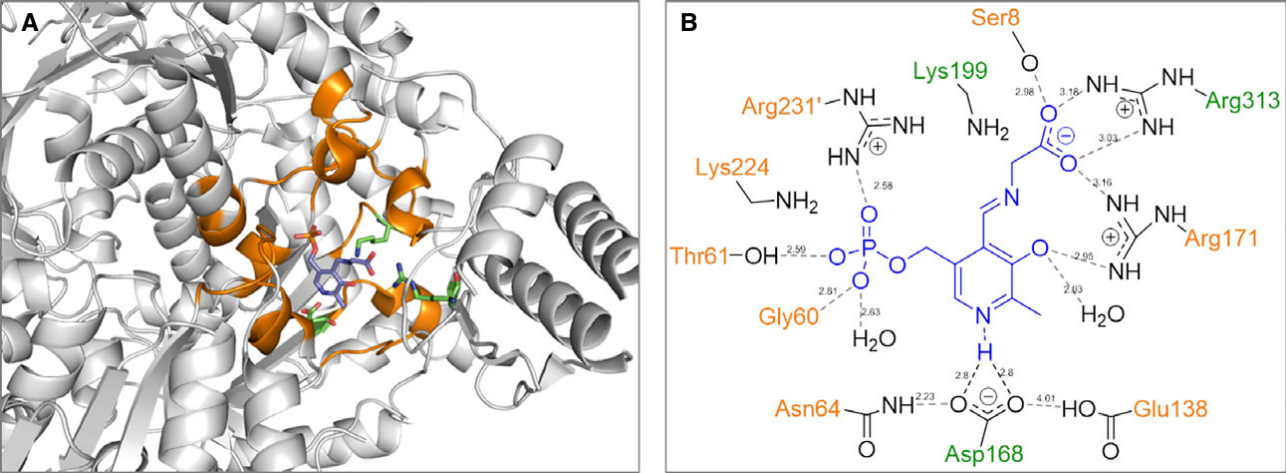
Structure of LTA from *Aeromonas jandaei* (PDB code PDB:3WGB): (A) family‐specific (orange) and conserved (green) positions in the enzymes of aspartate aminotransferase superfamily are shown in subunit A of the homotetramer. (B) Interaction of PLP with family‐specific (orange) and conserved (green) positions in the active site.

Bioinformatic analysis of sequence/structure‐function relationship in Fold type I PLP‐dependent enzymes (i.e., the aspartate aminotransferase superfamily) was carried out with a particular focus on LTA. Multiple alignment of a representative set of 9138 proteins with high structural, but low sequence similarity to LTA from *A. jandaei* was constructed by the Mustguseal and further analysed by the Zebra on‐line methods (see the Bioinformatic analysis of the aspartate aminotransferase superfamily in Experimental section). This representative set of homologs was automatically clustered into groups by maximizing sequence conservation within the groups and sequence variability between the groups. Further analysis of the available functional annotation of members in each predicted cluster revealed that they correspond to 15 functional families with different reaction and substrate specificities within the aspartate aminotransferase superfamily (Table 1). In particular, proteins annotated as LTAs in the PDB and Swiss‐Prot databases were classified into two functional families, what is in agreement with suggested convergent evolution of the LTA genes – one family comprises the LTAs highly specific towards l‐*allo*‐threonine, whereas another family contains low‐specificity LTAs which accept both l‐ and l‐*allo*‐threonine as substrates 24. The multiple alignment and the obtained annotation of functional families were used to identify conserved and FSPs in protein structures (Fig. 3, Table 1). Highly conserved positions in a functionally diverse superfamily define general properties common among all homologs, e.g., are directly involved in the enzyme catalytic machinery 18. On the contrary, the positions conserved only within functional families, but different between them, are defined as the ‘family‐specific positions’ or FSPs and can help to understand the molecular mechanisms of functional diversity, substrate and reaction specificity 13, 17, 18. The positions identified by bioinformatic analysis were further implemented as hotspots for site‐directed mutagenesis of LTAaj to study their implication to the catalytic activity and reaction specificity.

**Table 1:** Conserved and FSPs in the aspartate aminotransferase superfamily. One column of the table represents one column of the multiple alignment of the aspartate aminotransferase superfamily enzymes. N/A, no equivalent (i.e., a gap in the multiple alignment)

Functional family	PDB^a^	Conserved positions			FSPs											
l‐allo‐threonine aldolase	3wgb	D168	K199	R313	G60	T61	N64	H85	E138	A170	R171	C196	S198	G200	K224	R231
Serinehydroxymethyltransferase	2w7e	D197	K226	R357	G94	A95	N98	H122	A171	A199	H200	T223	H225	T227	Q255	Y51
2‐amino‐3‐ketobutyrate‐CoA ligase	3a2b	D210	K244	R367	G113	F114	N117	H138	D181	A212	H213	T241	S243	S245	F272	N85
Cystathionine β‐lyase	1ibj	D253	K278	R440	G157	M158	L161	Y181	E224	S255	I256	S275	T277	F279	N307	R129
Threoninephosphate decarboxylase	1lkc	D185	K216	R337	E85	T86	I89	F108	C153	A187	F188	S233	T215	F217	M244	Y56
l‐*low specificity*‐threonine aldolase	1v72	D174	K207	R327	G64	T65	N68	H89	T143	S181	R177	G204	T206	N208	K237	Y35
Alanine‐glyoxylate aminotransferase	2bkw	D175	K201	R354	G68	T69	W72	F97	T144	V177	C178	A198	Q200	A202	N/A	Y255
Tryptophanase	2ez2	D214	K257	R404	G99	R100	E103	F123	A171	T216	R217	S254	K256	D258	V283	Y71
Tyrosine aminotransferase	4ix8	D253	K286	R422	V150	S151	I154	F175	I171	I255	Y256	G283	A285	Y287	Q319	Y109
Kynureninase	1qz9	D201	K227	R375	T97	S98	L101	F129	T172	A203	H204	C224	Y226	Y228	N/A	Y226
Amine aminotransferase	4ba5	D259	K287	V423 (R416)	G120	S121	V124	Y153	E 226	V261	I262	T284	A286	G288	H318	K288
Acetylornithine aminotransferase	4ade	D222	K252	R374	G105	A106	N109	F138	E190	V225	Q226	T249	A251	A253	N/A	T281
l‐tyrosine decarboxylase	3f9t	D206	K245	R371	G94	T95	N98	H132	I177	A208	F209	D242	H244	M246	N/A	T285
Aspartate aminotransferase	4dbc	D223	K258	R386	G115	T116	L119	W142	H190	A225	Y226	S255	S257	N259	R292	N/A
Protein MalY aminotransferase	1d2f	D201	K233	R365	V96	I97	V100	Y121	C169	I203	H204	S230	S232	S234	K261	Y61

a The conserved positions and FSPs are provided for representative proteins in each functional family (the pdb field).

### Conserved positions K199, D168, R313 in the aspartate aminotransferase superfamily

Three residues K199, D168, and R313 (the residues numbering hereafter is given for LTAaj, PDB code PDB:3WGB, unless stated otherwise) were shown to be invariant (i.e., occupied by only one amino acid type) in all proteins used to build the aspartate aminotransferase superfamily alignment (Table 1), except for omega‐transaminase from *Chromobacterium violaceum* and its homologs, which have R313 substituted for V423 with R416 available in analogous orientation instead (PDB:4ba5). The selected positions are known to be important for the catalytic activity and participate in the cofactor and substrate binding in all enzymes of the aspartate aminotransferase superfamily.

The conserved K199 is a key catalytic residue located on the *si*‐face of the cofactor which forms the internal aldimine and participates in the formation of the external aldimine in all Fold type I PLP‐dependent enzymes 25. This residue also functions as a general base to deprotonate an amino acid substrate at C_α_ during the first step of the reaction, or as an acid to protonate C_4_′ of PLP in the quinonoid intermediate in many reactions catalysed by the Fold type I PLP‐dependent enzymes (Fig. 2) 11, 26. However, in LTAaj K199 does not participate in the C_α_‐proton abstraction in the external aldimine, as this role is performed by a base (presumably a water molecule) from the *re*‐side of PLP (opposite from K199) 27. Thus, K199 must be kept away from the C_α_ and C_4_′ atoms in the quinonoid intermediate to avoid its participation in the protonation/deprotonation step and to minimize competing reactions. On the other hand, K199 must attack the C_4_′ atom of the external aldimine to regenerate PLP and to release the reaction product. Therefore, the enzyme discriminates structures of the quinonoid intermediate and the external aldimine and changes the position of the side chain of K199 accordingly. This can be regulated by the mobility of a loop where K199 is located, which is trapped by the electrostatic interactions and H‐bonding with family‐specific amino acid residues S8, D9, C196, and S198. The mutation K199R reduces the specific activity of LTAaj by 5000 fold, which can be partially restored by adding an excess of PLP to the reaction medium (Table 2). This confirms the importance of the K199 residue in the binding of the cofactor and in the overall reaction mechanism of LTAaj.

**Table 2:** Kinetic parameters of the LTAaj's mutants for the retro‐aldol cleavage of l‐*allo*‐threonine and l‐β‐phenylserine. Conversion in the aldol reaction of glycine and benzaldehyde to synthesize l‐β‐phenylserine

Mutant	l‐*allo*‐threonine			l‐β‐phenylserine			
	*k*_cat_, s^−1^	*K*_m_, mm	*k*_cat_/*K*_m_, s^−1^·m ^−1^	*k*_cat_, s^−1^	*K*_m_, mm	*k*_cat_/*K*_m_, s^−1^·m ^−1^	Aldol reaction, conv. %^a^
wt	8.67	0.5	1.7 × 10^4^	60.5	3.7	1.6 × 10^4^	25 (*syn*)
K199R^b^	0.08	2.7	30	n.d.	n.d.		0
D168V	0.02	2.7	6.7	0.6	1.3	4 × 10^2^	20 (*syn*)
D168S^b^	0.42	1.2	350	0.83	4.1	2 × 10^2^	22 (*syn*)
D168N	0.05	2.6	20	n.d.	n.d.		20 (*syn*)
R313H	2.3	28	80	0.32	5.2	62	21 (*syn*)
R171F	0.23	18	10	0.17	7	24	0
R171Q	0.47	69	6.8	0.2	17	11	< 5
C196T	13.3	0.5	2.6 × 10^4^	84	3.7	2.2 × 10^4^	25 (*syn*)
S198H	5.1	5.7	0.9 × 10^3^	4.3	4.8	9 × 10^2^	17 (*syn*)
H85F	0.05	11.5	4.5	0.3	4.6	63	9 (*anti*)
H85Y	0.01	14.8	0.7	0.2	3.8	50	7 (*anti*)
R231A	0.7	7.5	90	2.3	4.1	6 × 10^2^	20 (*syn*)
N64E	0.3	5.7	60	0.08	1	80	26 (*syn*)
N64I	1	3.8	3 × 10^2^	0.2	0.8	3 × 10^2^	25 (*syn*)
G200S	13.6	0.2	6.1 × 10^4^	74.4	2.1	3.5 × 10^4^	24 (*syn*)
E138A	3.9	2.3	1.7 × 10^3^	1.9	1.2	1.6 × 10^3^	12 (*syn*)

a Reaction conditions: 50 mm benzaldehyde, 0.5 m glycine, 1 mg LTA, pH 8.0, 25°C, 24 h. *e.e*. > 99%; *d.e*. = 25–35% (the major isomer is given in parentheses).

b After addition of PLP.

**Figure 4:**
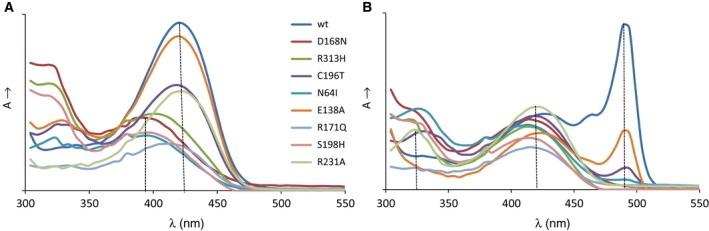
Formation of the (A) internal aldimine and (B) external aldimine with the glycine substrate in the wild type LTAaj and its mutants at pH 8 (λ_max_ = 340 nm corresponds to *gem*‐diamine; λ_max_ = 390 nm – free PLP; λ_max_ = 410–420 nm – internal/external aldimine; λ_max_ = 495 nm – quinonoid complex).

The conserved residue D168 in LTAaj is hydrogen‐bonded with the pyridine nitrogen N1 of PLP, keeping it protonated. The formation of the pyridinium cation induces resonance and the electron sink effect to delocalize the carbanion charge in the cofactor ring and yield a stable quinonoid form (Fig. 2). Formation of the long‐lived quinonoid intermediate enhances the rate of catalysis in the enzymes of aspartate aminotransferase superfamily 28. As was noted before, the contribution of N1 protonation to the stabilization of carbanions generated in the aldol processes is not as crucial as in the transamination process 28. A mutation of D168 to Thr, Ser, Val, or Asn in LTAaj greatly reduced the specific activity, which was partially restored by the addition of excess PLP to the reaction mixture (Table 2). Despite this, the mutant enzyme variants were able to catalyse the aldol synthesis of l‐β‐phenylserine from benzaldehyde and glycine with yields similar to the wild‐type enzyme. Further, the protonation of pyridine nitrogen N1 by the aspartate residue may influence the electrophilicity of the cofactor. The D168 mutants in LTAaj did not form the internal aldimine (λ_max_ = 410 – 420 nm, Fig. 4A, red line), whereas formation of the external aldimine was observed when using the glycine substrate (λ_max_ = 420 nm, Fig. 4B red line). Thus, the mutation at D168 jeopardizes the nucleophilic attack of the ε‐amino group of K199 on the aldehyde group of the cofactor due to diminished electrophilic properties of PLP. To summarize, D168 in LTAaj is important for the correct binding of the PLP cofactor in the active site and the regulation of the electron‐withdrawing capacity of the cofactor. The disruption of hydrogen bonding with N1 of PLP is not lethal for the aldol reactions catalysed by LTAaj, however significantly reduces the rates of a catalytic conversion.

The conserved R313 in LTAaj acts as a α‐carboxylate docking site for the amino acid substrates 29, 30. Similar to other Fold Type I PLP‐dependent enzymes, the electrostatic interaction between the carboxylic group of the α‐amino acid substrate and R313 in LTAaj may stabilize the Michaelis complex and maintain pKa of the C_α_ atom 31. The catalytic activity of the mutant R313H in the retro‐aldol cleavage of l‐*allo*‐threonine was decreased by 200 fold mainly due to increase in *K*_m_ (Table 2). Furthermore, the mutation R313H hinders the formation of the quinonoid complex to stabilize the carbanion after initial bond cleavage (λ_max_ = 495 nm, Fig. 4B, green line). Nevertheless, l‐β‐phenylserine product in the aldol condensation was obtained with the conversion rate similar to the wild‐type enzyme (Table 2). These data confirm the multiple roles of R313 as an important site for binding and accommodation of the amino acid substrate to enhance the acidity of the C_α_ atom and promote the initial bond cleavage in LTAaj.

### Family‐specific positions in the aspartate aminotransferase superfamily

**Figure 5:**
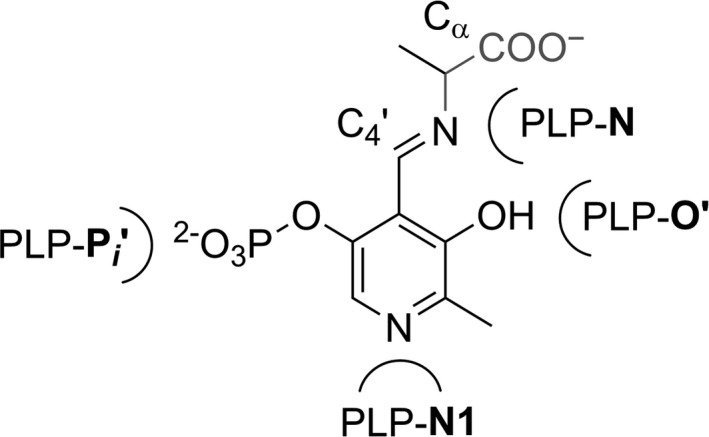
The nomenclature of functional groups of PLP cofactor used in the manuscript.

The bioinformatic analysis has shown that each functional family within the aspartate aminotransferase superfamily can be characterized by a unique signature pattern of FSPs that can determine the observed functional variability among related enzymes (Table 1). The most statistically significant FSPs identified by the bioinformatic analysis were located in the cofactor‐binding site of homologous proteins (Fig. 3). These FSPs participate in the specific hydrogen‐bonding and electrostatic interactions with functional groups of PLP (N, N1, O’, Pi, Fig. 5) thus influencing the properties of the cofactor to establish the catalytic reactivity and reaction preference.

#### The family‐specific positions G60, T61, H85, A170, R231

The phosphate group binding subsite has a common recognition motif within the functional families diversified by the FSPs. In the active site of LTAaj, the phosphate group interacts with G60, T61, R231, and a water molecule, which defines the flexibility of the cofactor in the active site 30.

**Figure 6:**
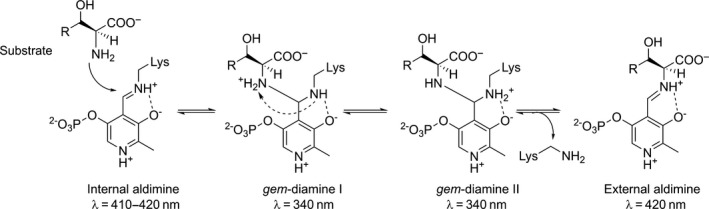
Formation of the external aldimine via transimination reaction in the active site of the Fold type I PLP‐dependent enzymes.

In addition to stabilizing the phosphate group of PLP, the family‐specific residue R231 accompanied by Y32 can participate in the proton transfer during formation of an external aldimine in LTAaj. The R231 can mediate the protonation of ε‐amine group of the Schiff base‐forming K199 similarly to serine hydroxymethyltransferases, where the equivalent residue at this FSP (i.e., Y51 in PDB:2W7E, Table 1) acts as a general acid‐base in the proton transfers between *gem*‐diamine I and II that occur during the external aldimine formation (Fig. 6) 32, 33, 34. This suggestion is in agreement with the experimental data – the strong absorption band at 340 nm was obtained for R231A mutant, which can indicate the formation and accumulation of *gem*‐diamine intermediate (Fig. 4B light green line). In addition, the R231A mutation hinders the formation of quinonoid species with the glycine substrate (495 nm, Fig. 4B), thus reducing the retro‐aldol catalytic activity of the LTAaj variant by 200 fold with the l‐*allo*‐threonine substrate (Table 2) and confirming the role of this residue in catalysis.

**Figure 7:**
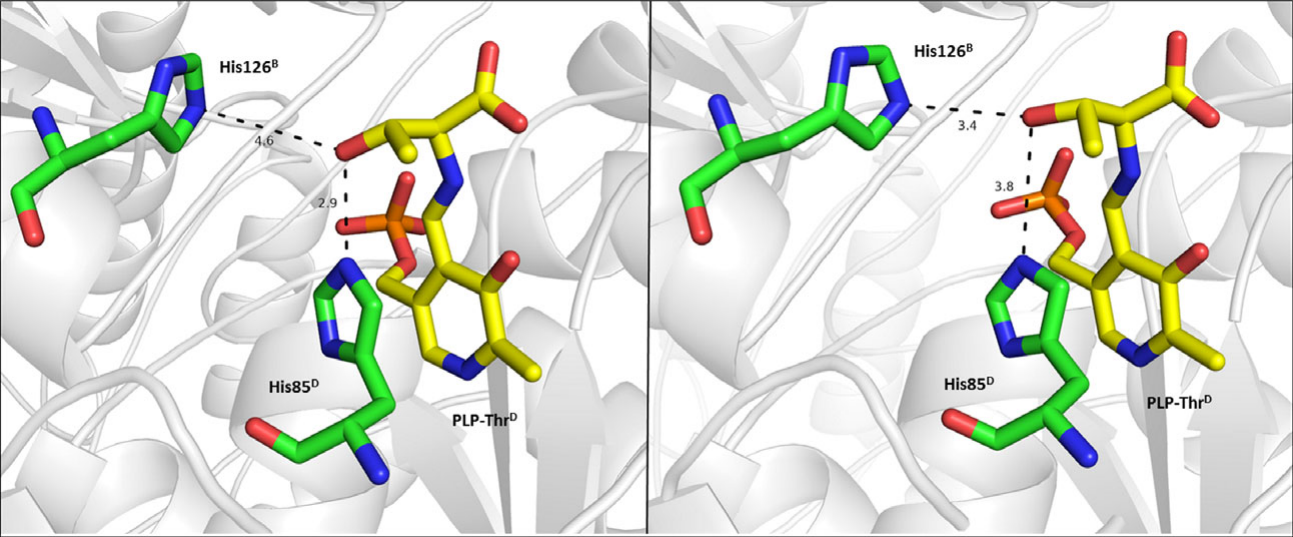
Molecular dynamics snapshot of the LTA from *Aeromonas jandaei* with the l‐allo‐threonine substrate. The family‐specific residue His85 and His128 take turns in accommodating the OH‐group of the l‐*allo*‐threonine during the MD simulation (MD snapshots are taken at A: 15 ns; B: 40 ns).

The cofactor binding site is located at the bottom of the enzyme active center so that the pyridine ring interacts with the family‐specific residue A170 on the *si*‐face of the PLP ring in LTAaj. A small aliphatic residue at this position is common for aspartate aminotransferase superfamily (Table 1) and presumably restricts the rotation of the PLP ring. On its *re*‐face, the cofactor interacts with the family‐specific residue H85 in LTAaj, which is occupied by an aromatic residue in other homologs and is parallel to the PLP ring. The aromatic residue at this position increases the electrophilic character of the conjugated PLP system 35. The FSP H85 together with residue H128 in LTAaj may act as a catalytic base for the proton abstraction from C_β_‐OH of an l‐β‐hydroxy‐α‐amino acid substrate to initiate the retro‐aldol cleavage 30. Indeed, the mutations H85F and H85Y reduced *k*_cat_/*K*_m_ by 4000 fold in the retro‐aldol reaction with the l‐*allo*‐threonine substrate (Table 2). The formation of quinonoid intermediates with a glycine substrate in the H85F and H85Y mutants was not observed, and the conversion in the aldol reaction was only 7% and 9% after 24 h, respectively. Both mutants H85Y and H85F changed the stereoisomer preferences in the aldol condensation of glycine and benzaldehyde to make preferably *anti*‐isomer of l‐β‐phenylserine, whereas the *syn*‐isomer was the major product in the reaction with wild‐type LTAaj (Table 2). LTAs are known to be highly selective at C_α_ but moderately selective at C_β_ of the β‐hydroxy‐α‐amino acid substrates 36. Recently, H85 and H128 were suggested to play an important role in the determination of C_β_‐stereospecificity of the LTAaj, when both can serve as an acid for the re‐protonation of the C_β_‐oxygen in the aldol reaction 30. Until now, attempts to alter the stereoselectivity of LTAs at the β–carbon in the aldol synthesis of unnatural substrates by enzyme engineering have failed mainly due to complex hydrogen bonding network in the active center related to the protonation step 9. The molecular dynamics simulation of LTAaj homotetramer showed that OH‐group of the l‐*allo*‐threonine can form a hydrogen bond to either H85 or H128 and the two histidines take turns in accommodating the substrate by changing the orientation of their rotamers (Fig. 7).

#### The family‐specific position R171

**Figure 8:**
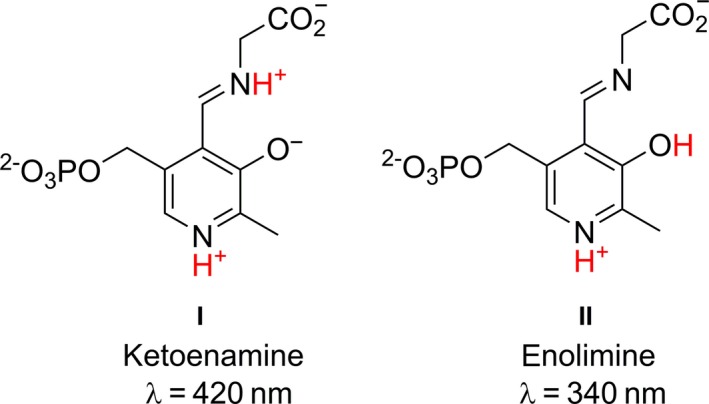
PLP‐glycine Schiff base tautomers.

**Figure 9:**
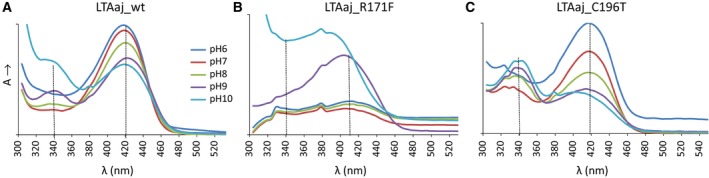
Coenzyme absorbance spectra obtained in the pH titration of the wild‐type LTAaj and its mutants. λ_max_ = 340 nm – enolimine; λ_max_ = 420 nm – ketoenamine. (A) wild type LTAaj; (B) R171F mutant of LTAaj; (C) C196T mutant of LTAaj.

The FSP R171 in LTAaj is hydrogen‐bonded to the phenolic oxygen O’ of the PLP and may be important to maintain the tautomeric equilibrium of PLP aldimines. This is controlled by the position of a bridging proton, which is part of an intramolecular hydrogen bond (PLP‐O’··H··N‐PLP), to undergo a proton transfer from *N*‐protonated ketoenamine (Fig. 8, I) to *O*’‐ protonated enolimine PLP tautomer (Fig. 8, II). We have investigated the tautomer equilibrium in the active center of LTAaj using a spectrophotometric approach. The ketoenamine isomer I has an absorption maximum at 420 nm and the enolimine isomer at 340 nm. At least two ionization states were observed in the LTAaj coenzyme absorbance spectra taken as a function of pH (Fig. 9A). The formation of absorption species corresponding to the enolimine II was observed at pH ≥ 9, indicating that at physiological pH the imine nitrogen N of PLP is fully protonated (ketoenamine tautomer) in LTAaj.

To select residues with the largest impact on the tautomeric equilibrium in LTAaj, we have performed *in silico* mutagenesis using FSPs as hotspots and compared the imine's pKa in the structures of different mutants (Table 3). Introduction of mutations at FSPs R171, R313 and H85 resulted in a significant decrease of the imine's acidity, with the largest increase of pKa by 2 units observed for the R171F mutant. On the contrary, substitutions at positions C196, D9, and D168 increased the acidity of the imine. The selected mutants, which were shown by the molecular modelling to have the largest influence on the imine's pKa, were produced *in vitro* and their absorption specters as a function of pH were studied. The formation of the enolimine tautomer for the R171F mutant was not observed up to pH 9.5 (higher pH caused denaturation of the enzyme; Fig. 9B), whereas the enolimine tautomer was observed for the C196T mutant already at pH ≥ 7 (Fig. 9C). The disruption of hydrogen bonding between O’ of PLP and R171 caused by the R171F mutation resulted in the loss of specific activity by more than 2000‐fold (Table 2). Similar to the previously discussed mutations at the conserved positions D168N and R313H, and the FSP H85F, a quinonoid species for the PLP‐glycine complex was not obtained for the R171F mutant (Fig. 7B, grey line), confirming the essential role of these residues in the carbanion stabilization. Moreover, R171F was the only tested mutant which was not active in the aldol condensation reaction to produce l‐β‐phenylserine. It can be concluded that the FSP R171 has a significant impact on the tautomeric equilibrium in LTAaj, which controls the acidity of the imine nitrogen N (and the C_α_ of a substrate) in the external aldimine complex. Its mutation hinders the initial bond cleavage and inactivates the enzyme.

**Table 3:** The *in silico* estimation of the pKa values of the PLP's imine and ε‐amino group of K199 in the mutants of LTAaj

Mutation	pKa PLP‐N	Mutation	pKa PLP‐N	Mutation	pKa ε‐NH2 K199
D9A	8.38 ± 0.82	D9A/C196T	7.91 ± 0.76	C196T	9.87 ± 0.61
C196T	8.47 ± 0.8	H85F/D168S	8.22 ± 0.8	D9A	11.57 ± 0.92
D168S	8.48 ± 0.83	D168S/G200F	8.22 ± 0.77	S198H	11.65 ± 1.01
H85F	8.63 ± 0.79	E90A/D168T	8.23 ± 0.75	D168S	11.66 ± 0.9
E90A	8.67 ± 0.82	C196T/G200S	8.23 ± 0.75	H85F	12.04 ± 1.06
S198H	8.78 ± 0.72	D168S/S198H	8.34 ± 0.7	E138A	12.05 ± 1.06
G200S	8.8 ± 0.81	E90A/E138A	8.41 ± 0.88	wt	12.07 ± 1.04
E138A	8.86 ± 0.84	N64E/D168N	8.61 ± 0.77	N64E	12.13 ± 1.11
wt	8.89 ± 0.83	wt	8.89 ± 0.83	R313H	12.17 ± 1.07
N64E	8.99 ± 0.81	D168S/R313H	9.25 ± 0.68	R171F	12.78 ± 1.42
H85Y	9.21 ± 0.85	H85Y/R171F	11.25 ± 1.3	R231A	12.85 ± 1.52
R313H	9.7 ± 0.77	D9A/R171Q	11.28 ± 1.81	R171Q	12.93 ± 1.5
R171F	10.42 ± 0.84	R171F/R313A	11.42 ± 0.69		
R171Q	10.63 ± 0.82	R171Q/R231A	12.51 ± 1.72		

#### The family‐specific positions C196, S198, G200

The FSPs C196, S198, and G200 are located in the loop which hosts the conserved Schiff base‐forming K199 in LTAaj. The positions around the K199 are specific in all functional families of the aspartate aminotransferase superfamily (Table 1). In particular, the family of l‐*allo*‐threonine aldolase is characterized by the C196‐X‐S198‐K199‐G200 sequence pattern. To avoid participation of K199 as the acid/base catalyst in the LTAaj's reactions and eliminate undesirable side‐effects (e.g. deprotonation at C_α_ from the *si*‐side or protonation at C_4_′), its nucleophilicity has to be controlled. According to *in silico* calculations the C198T and S198H mutations decrease the pKa of ε‐amino group of K199 by 2 units (Table 3) and the C196T mutation decreases the pKa of the imine nitrogen by 0.5 units, what correlates with the experimental data. The produced C196T mutant has a prominent absorption band at 340 nm at pH ≥ 7, which corresponds to the enolimine tautomer of the internal aldimine (Fig. 9C), indicating a change of imine's pKa. To conclude, the family‐specific residues C196, S198 and G200 apparently influence the nucleophilic properties and mobility of the side‐chain of Schiff base‐forming lysine K199, can participate in a proton transfer to/from its ε‐amino group and in the regulation of imine's pKa (and, thereafter, pKa of C_α_ of the substrate in the external aldimine).

#### The family‐specific positions N64, E138

As discussed above, protonation of pyridine nitrogen N1 of PLP via a conserved aspartate (D168 in LTAaj) is an important mechanism to reduce the pKa of N1 and activate the cofactor as an electrophile in aspartate aminotransferase superfamily enzymes. However, most often a single carboxylic group of Asp is not sufficient to decrease N1's pKa 37. In LTAaj D168 is hydrogen bonded with the family‐specific residues N64 and E138, which assist the conserved aspartate in a pyridine nitrogen protonation. Upon mutations N64I and E138A in LTAaj, no internal aldimine was formed and the specific activity was reduced by more than 500 fold, thus having a similar effect as D168T mutation (Fig. 4A, light blue line). This finding provides evidence that the specific microenvironment near pyridine N1 of PLP and D168 indirectly influences the protonation state of pyridine nitrogen and thus modulates electrophilicity of the cofactor affecting the rate of catalytic conversion.

### Evolved promiscuous activities

Many PLP enzymes are known to possess catalytic promiscuity which can be modulated by point mutations 38. E.g., recently it was shown that the bacterial alanine racemase can be converted to d‐threonine aldolase by a single mutation 39, the decarboxylase activity can be implemented in the aspartate aminotransferase 40 and vice versa 41. As concluded above, the FSPs in LTAaj's structure create an active site microenvironment that accommodates a particular protonation state of the cofactor to regulate the reaction specificity, and thus can be used as hotspots to design novel enzymes. According to the previous studies, the protonation state of pyridine nitrogen N1 and phenolic oxygen O’ of PLP modulates the carbon acidity of C_α_ and C_4_′, and is one of the main factors determining the reaction path in the PLP‐dependent enzymes 42. Proceeding from the relative acidities of C_α_ and C_4_′, the enzyme defines at which carbon atom the protonation should occur in the quinonoid intermediate to release the product (Fig. 2). As it was shown above the reaction specificity in LTAaj is also controlled by modulating the nucleophilicity of the conserved K199. Thus, modification of the microenvironment around the cofactor and K199 can change both the substrate specificity and the reaction preference.

**Figure 10:**
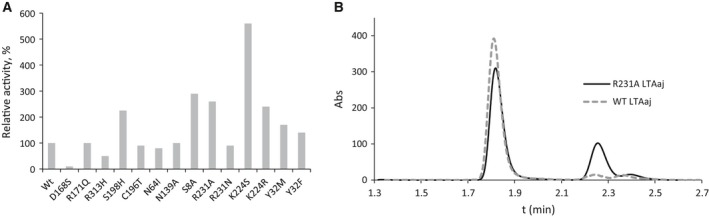
(A) Transaminase activity in the mutants of LTA from *Aeromonas jandaei* with respect to wild type (100% corresponds to the transaminase activity in WT 0.6 U·mg^−1^). (B) Racemization of l‐alanine to d‐alanine catalysed by R231A LTAaj (black line) and wild type LTAaj (dashed grey line). After 8 h: *e.e*.(WT) = 99%, *e.e*.(R231A) = 46%.

**Figure 11:**
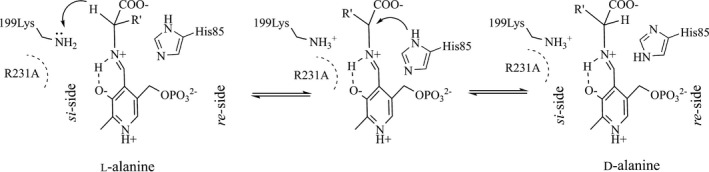
Proposed mechanism of l‐alanine racemization catalyzed by R231A LTA from *Aeromonas jandaei*.

We performed mutagenesis of the selected FSPs in LTAaj and evaluated the produced variants for new catalytic activities. Mutations at positions close to C4′ and K199 increased the promiscuous transaminase activity up to 6 fold in LTAaj (Fig. 10A), and a substitution of the family‐specific residue R231 introduced a novel alanine racemase activity and reduced the native aldol activity of the enzyme. The mutation R231A was shown to increase the pKa of ε‐amino group of K199 (Table 3) and consequently the K199's nucleophilicity to act as a base on the *si*‐side of PLP (Fig. 11). Thus, l‐alanine was racemized to d‐alanine by the R231A mutant, whereas the wild‐type enzyme was not active towards l‐alanine (Fig. 10B). To conclude, the promiscuous activities were evolved in LTAaj by introducing point mutations at the selected FSPs which were shown to be able to modulate the capacity of the ε‐amino group of K199 to act as a second base thus directing the racemization or the transamination of the substrate.

## Conclusion

The freely available on‐line methods Mustguseal and Zebra were used to perform a systematic bioinformatic analysis of the aspartate aminotransferase superfamily proteins, which share a common structural framework but implement different catalytic functions, to identify the conserved amino acid residues responsible for the common properties of diverse homologs, as well as the FSPs – determinants of functional variability. It was further shown by experimental site‐directed mutagenesis and molecular modelling that the FSPs create a specific microenvironment around the cofactor in the active center of LTA from *A. jandaei* to regulate the protonation state of PLP's functional groups and ultimately modulate its electrophilicity to optimize the retro‐aldol catalytic reaction pathway thus minimizing the unwanted side reactions. The FSPs H85, R171, and conserved residue R313 directly affect the acidity of imine and α‐carbon in the external aldimine, hinder the efficient carbanion stabilization and have the largest impact on the catalytic activity of the LTAaj. Moreover, a specific protonation state of external aldimine – controlled by the FSPs R171, K224, R231 and by the conserved residue D168 – favours a negative charge at C_α_ after retro‐aldol C_α_–C_β_ bond cleavage of the β‐hydroxy‐α‐amino acid substrate and directs proton transfer to the C_α_, what is followed by the glycine product release. The concomitantly formed positive charge at C_4_’ of PLP helps to maintain the reaction specificity by disfavouring the competing pathways (e.g., transamination). The obtained results shed additional light on the role of proton transfer in LTAaj's catalysis and support the hypothesis that reaction specificity in PLP‐dependent enzymes is settled in part by the tautomeric equilibrium of ionisable groups of PLP and the reacting substrates 11, 43. The extent of PLP activation as an electrophilic catalyst is regulated not only by protonation of the pyridine's nitrogen of a cofactor through a conserved D168, but also by the local microenvironment surrounding this aspartate, i.e. the family‐specific residues N64 and E138. On the other hand, the phenolic oxygen protonation influenced by R171 can have a similar or even a greater effect on the cofactor activation to delocalize the electrons in the carbanion intermediate. The nucleophilicity and mobility of the side chain Schiff base‐forming K199 are regulated by the surrounding family‐specific residues C196, S198, K224, and R231, which were shown to play an important role in controlling the reaction specificity. Consequently, by changing this specific microenvironment the natural activity of LTAaj can be decreased and the promiscuous reactions can be induced; thereby racemase and transaminase activities have been evolved by mutating one of FSPs, e.g. R231, K224, S198, in the active site of LTAaj.

The bioinformatic analysis of conserved and FSPs in the aspartate aminotransferase superfamily was used to identify key residues important for the retro‐aldol and aldol transformations and the reaction specificity in LTA from *A. jandaei*. The revealed FSPs are conserved within functional families of the Fold type I PLP‐dependent enzymes, and different between them, and thus can determine the reaction pathways not only in the LTAaj enzyme but in the entire aspartate aminotransferase superfamily. The implication of these positions to the functional and catalytic diversity in other Fold type I PLP‐dependent enzymes should be further investigated to establish the structure‐function relationship and design novel enzymes for biotechnological applications. The obtained results provide insight into the structural basis of catalytic promiscuity of the PLP‐dependent enzymes and demonstrate the potential of bioinformatic analysis at studying structure‐function relationship in protein superfamilies using freely available on‐line resources.

## Experimental

### General

All chemicals were purchased from Sigma‐Aldrich (St. Louis, MO, USA) or Alfa Aesar (Karlsruhe, Deutschland), unless stated otherwise. All enzymes for the genetic work were purchased from ThermoFisher Scientific (Waltham, MA, USA). The expression vector pEamTA was produced as stated before 44. The *Escherichia coli* strain BL21(DE3) was obtained from BioLabs (New England, MA, USA). Analytical HPLC was carried out with an Agilent 1100 HPLC system (Santa Clara, CA, USA) equipped with a G1315A diode array detector. A Chromolith Perfomance RP‐18e (100 × 4.6 mm; Merck Millipore, Merck KGaA, Darmstadt, Germany) column was used for analysis. Phenylserine isomers were determined by HPLC after derivatization with *ortho*‐phthaldialdehyde/*N*‐acetyl cysteine 36. UV‐vis scans and single wavelength kinetics were measured on a Perkin Elmer Lambda 2 spectrophotometer (Waltham, MA, USA).

### Bioinformatic analysis of the aspartate aminotransferase superfamily

The multiple structure‐guided sequence alignment of the PLP‐dependent enzymes of the Fold type I was constructed using the Mustguseal web‐server (https://biokinet.belozersky.msu.ru/mustguseal) 22. Mustguseal implements a recently described protocol 45 which is based on a combination of protein sequence and structure comparison algorithms to account for structural and functional variability within a large superfamily. First, comparison of protein structures was implemented to study distant evolutionary relationships because structures are more conserved in evolution than sequences. The PDB structure PDB:3WGB of the l‐*allo*‐threonine aldolase from *A. jandaei* was used as a query for a structure similarity search in the PDB database to collect a non‐redundant set of 15 proteins which shared 67–91% secondary structure equivalences (8–20% pairwise sequence similarity) with LTAaj and represented families of aspartate aminotransferase superfamily with different reaction and substrate specificities. The missing loop regions in PDB structures were reconstructed using the Modeller software 46. These 16 representative proteins (including the LTAaj) were superimposed to build the core structural alignment. Then, each representative protein was independently used as a query for a sequence similarity search in Swiss‐Prot and TrEMBL databases to collect its evolutionary close relatives – members of the corresponding families (at most 1000 sequences per search). The obtained sequence sets were further filtered to remove redundant entries (at the 95% pairwise sequence identity threshold) and outliers (which shared < 0.5 bit score per column or differed in length by more than 20% with the corresponding representative protein) within each family, and superimposed using the core structural alignment of the representative proteins as a guide. The final structure‐guided sequence alignment contained 9138 sequences and structures of PLP‐dependent enzymes of the Fold type I (aspartate aminotransferase superfamily) with high structural, but low sequence similarity to LTAaj and characterized by different reaction specificities. The proteins were automatically classified into the specificity groups 17 which corresponded to the families with different reaction specificities within the aspartate aminotransferase superfamily. The constructed alignment and the proposed functional classification was further operated by the Zebra web‐server (https://biokinet.belozersky.msu.ru/zebra) to automatically select the statistically significant conserved and FSPs in protein structures 23.

### *In silico* pKa estimation

The molecular dynamics simulation was used to generate an ensemble of structural variants of the wild‐type LTAaj which were then implemented as template structures to construct a set of molecular models of LTAaj's mutants. The pKa values of the imine of PLP and ε‐amino group of K199 in the wild‐type protein and its mutants were estimated as an average of the respective pKa values predicted in each structural conformer.

The modified version of PDB2PQR v. 1.9.0 47 compatible with propka v. 3.1 48 was implemented to build a model of the tetramer of l‐*allo*‐threonine aldolase from *A. jandaei* based on the PDB structure PDB:3WGB. ambertools v.15 (San Francisco, CA, USA) package was used to parametrize the protein in the FF14SB Amber force field. The quantum chemistry package pcgames/firefly v. 8.1.1 and R.E.D. v. III.52 package 49 were used to optimize the geometry of the external aldimine and calculate its atomic charges. Parameters for the phosphate moiety of the cofactor were taken from Steinbrecher *et al*. 50. The protein system was solvated in a cubic box using the TIP4P‐Ew water model so that the distance from any atom of the protein to the edge of the periodic cell was at least 12 Å. The final size of the protein system in a water box was ~ 139 000 atoms. The protocol for classical MD simulation was adapted from Pierce *et al*. 51. The initial system was energy minimized, heated from 0 K to 300 K in the NVT ensemble, and equilibrated in the NPT ensemble at 300 K and constant isotropic pressure of 1 atm. The density, temperature and total energy plots clearly converged by the end of the equilibration period. The obtained system was used for a free (unconstrained) run in the NVT ensemble at 300 K for 100 ns. Molecular dynamics calculations were performed using the GPU‐implementation of the explicit solvent PME simulation method in AMBER 52, 53. The minimization, heating, and equilibration steps of each simulation were carried out on a locally installed GeForce GTX 980 Ti GPU‐accelerator, and the final production run was executed in MPI mode on four Tesla K40 units of the supercomputer. An ensemble of structural variants of the wild‐type LTAaj were collected from the free (unconstrained) part of the MD trajectory within the period from 20 to 100 ns with a time step of 5 ns. Each structural variant of the protein was independently used as a template for molecular modeling of each mutant described in this manuscript using the mutate v. 2.0.1 software 54. For the wild‐type protein and each mutant the pKa values of the imine of PLP and ε‐amino group of K199 were estimated by propka v. 3.1 separately in all respective structural variants and then the average and standard deviation values were calculated. The construction and processing of mutant structural variants and the corresponding pKa calculation using the abovementioned software was carried out in parallel on a supercomputer using the mpiWrapper workflow manager 55.

### Cloning of LTAaj and site‐directed mutagenesis

The wild‐type LTAaj was cloned with a C‐terminal His‐tag as previously described 6. Site‐directed mutagenesis was performed on the wild‐type plasmid using complementary primers (IDT) and plasmid replication (Agilent, QuikChange II, Santa Clara, CA, USA). The plasmids were purified and then transformed into BL21(DE3) *E. coli* expression host cells.

### Proteins expression and purification

The constructs contained the *tac* promoter and the LacI repressor, which control the protein expression level. Three hundred milliliter 2xYT medium (in a 2 L flask) containing ampicillin (50 μg·mL^−1^) was inoculated with 5 mL of an *E. coli* overnight culture. Cells were grown at 37 °C to an OD_600_ of 0.6–0.8. Protein expression was induced by the addition of 1 mm IPTG and the culture was allowed to grow overnight at 20 °C. The biomass of the bacterial cultures was harvested by centrifugation at 4500 ***g*** for 10 min at 4 °C. The cell pellets were re‐suspended in sodium phosphate buffer (0.1 m, pH 7.4) and disrupted by ultrasonic treatment for 7 min. The crude lysate was cleared by centrifugation at 20 000 ***g*** for 1 h at 4 °C and subjected to further purification. Lysates were loaded onto a Ni‐NTA Superflow resin (Qiagen, Hilden, Germany), washed with sodium phosphate buffer (50 mm, pH 7.4) containing imidazole (5 mm) and NaCl (0.3 m) and subsequently eluted with imidazole (0.3 m) in sodium phosphate buffer (50 mm, pH 7.4). To remove the imidazole, the pooled and concentrated protein fractions were loaded on a Sephadex desalting column PD‐10 (GE Healthcare, Little Chalfont, UK) and eluted with sodium phosphate buffer (50 mm, pH 7.4) containing 0.1 mm PLP. The purified proteins were used for the activity measurements and biocatalytic transformations.

### Activity measurement towards l‐allo‐threonine

Threonine aldolase activity (cleavage of l‐*allo*‐threonine to acetaldehyde and glycine) was monitored using a coupled assay with alcohol dehydrogenase. The kinetic assays were performed in NaPi buffer (50 mm, pH 8) and contained of l‐*allo*‐threonine (0.1–100 mm), PLP (50 μm), NADH (200 μm), yeast alcohol dehydrogenase (30 U) and LTA (10–50 μm) in a final volume of 1 mL at 25 °C. NADH decrease was monitored photometrically at 340 nm (ε = 6.2 × 10^3^ m ^−1^·cm^−1^). Controls without the addition of TAs were carried out under the same conditions. The initial velocities were plotted and fitted to the Michaelis‐Menten equation in order to obtain the kinetic parameters. KaleidaGraph was used for data fitting.

### Activity measurement towards l‐phenylserine

The activity was verified by monitoring the formation of benzaldehyde upon cleavage of l‐*syn*‐β‐phenylserine (0.2–12 mm) at 279 nm (ε = 1.4 × 10^3^ m ^−1^·cm^−1^). The kinetic assays were carried out at 25 °C in NaPi buffer (50 mm, pH 8) containing PLP (50 μm) and LTA (10–50 μm).

### Spectroscopic analysis of LTAaj and variants

The spectral scanning of purified LTAaj and mutants was performed in 1 mL sodium phosphate buffer (50 mm, pH 8.0) with LTA (0.2 mg) in a 1 cm light path cuvette at the wavelength range of 300–550 nm at 22 °C. The formation of an external aldimine (λ = 420 nm) and quinonoid intermediate (λ = 495 nm) was monitored after addition of glycine substrate (0.1 m final concentration). Similar experiments were carried out over the pH range 6.0–9.5 to monitor accumulation of *N*‐deprotonated PLP enolimine tautomer (λ = 330 nm) and *N*‐protonated PLP ketoenamine tautomer (λ = 410–420 nm) and estimate the pKa of Schiff base nitrogen.

### Transaminase activity measurement

The activity towards transamination of d‐alanine was monitored using a coupled assay with lactate dehydrogenase. The kinetic assays were performed in sodium phosphate buffer (50 mm, pH 8) and contained d‐alanine (50 mm), PLP (50 μm), NADH (150 μm), lactate dehydrogenase (2.7 U) and purified LTA (50 μm) in a final volume of 1 mL at 25 °C. NADH decrease was monitored photometrically at 340 nm (ε = 6.2 × 10^3^ m ^−1^·cm^−1^).

### Racemization of l‐alanine

The enzymatic reactions were performed in 1 mL reaction volume in sodium phosphate buffer (50 mm, pH 8.0), containing PLP (50 μm) and l‐alanine (0.02 m) with wild‐type and mutant LTAaj (0.1 mg) at 25°C for 24 h. The formation of d‐alanine was monitored by RP‐HPLC after a pre‐column derivatization with Marfey's reagent (1‐fluoro‐2‐4‐dinitrophenyl‐5‐l‐alanine amide, λ_max_ = 338 nm) as described before 56. Eluent: H_2_O (0.1% HCOOH)/CH_3_CN = 50/50, 2.5 mL·min^−1^: *t* _l__‐ala_ = 1.77 min, *t* _d__‐ala_ = 2.26 min.

### General procedure for the synthesis of l‐β‐phenylserine

The enzymatic transformations were performed in 1 mL reaction volume in sodium phosphate buffer (50 mm, pH 8.0), containing PLP (50 μm), DMSO (10%, v/v), benzaldehyde (20 mm) and glycine (0.2 m). Transformations were performed at 30 °C with 0.1 mg of protein. After 24 h, 50 μL of the reaction mixture was diluted in 950 μL sodium tetraborate buffer (0.1 m, pH 10.5) and used for the determination of conversion by RP‐HPLC after a pre‐column derivatization as described before 36.

OPA/NAC derivatization: buffer KH_2_PO_4_ (20 mm, pH 6.8)/CH_3_CN = 86/14, 2.5 mL·min^−1^: *t* _l__‐syn_ = 2.7 min, *t* _l__‐anti_ = 4.1 min.

## Author contributions

The experiments were conceived and planned, and the manuscript was written by KF, DS, and VŠ; experiments were performed by KF and DS. The final version of this manuscript has been read and approved by all authors.
